# Variations in Spontaneous Assembly and Disassembly of Molecules on Unmodified Gold Nanoparticles

**DOI:** 10.1186/s11671-016-1615-2

**Published:** 2016-09-14

**Authors:** Ng Zhang Jin, Stanley Anniebell, Subash C. B. Gopinath, Yeng Chen

**Affiliations:** 1 School of Bioprocess Engineering, Universiti Malaysia Perlis, 02600 Arau, Perlis Malaysia; 2 Institute of Nano Electronic Engineering, Universiti Malaysia Perlis, 01000 Kangar, Perlis Malaysia; 3 Department of Oral Biology and Craniofacial Sciences, Faculty of Dentistry, University of Malaya, 50603 Kuala Lumpur, Malaysia; 4 Oral Cancer Research and Coordinating Center (OCRCC), Faculty of Dentistry, University of Malaya, 50603 Kuala Lumpur, Malaysia

**Keywords:** Gold nanoparticle, PEG-polymer, Factor IX, Factor IX-bp, Colorimetry

## Abstract

Electrostatic attraction, covalent binding, and hydrophobic absorption are spontaneous processes to assemble and disassemble the molecules of gold nanoparticles (GNP). This dynamic change can be performed in the presence of ions, such as NaCl or charged molecules. Current research encompasses the GNP in mediating non-biofouling and investigating the molecular attachment and detachment. Experiments were performed with different sizes of GNP and polymers. As a proof of concept, poly(ethylene glycol)-*b*-poly(acrylic acid), called PEG-PAAc, attachment and binding events between factor IX and factor IX-bp from snake venom were demonstrated, and the variations with these molecular attachment on GNP were shown. Optimal concentration of NaCl for GNP aggregation was 250 mM, and the optimal size of GNP used was 30 nm. The polymer PEG-PAAc (1 mg/ml) has a strong affinity to the GNP as indicated by the dispersed GNP. The concentration of 5800 nM of factor IX was proved to be optimal for dispersion of GNP, and at least 100 nM of factor IX-bp was needed to remove factor IX from the surface of GNP. This study delineates the usage of unmodified GNP for molecular analysis and downstream applications.

## Background

**Fig. 1:**
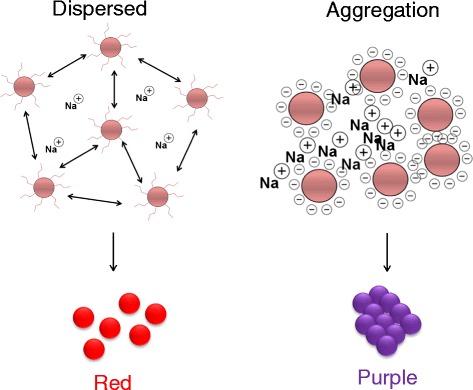
Mechanism for the process of aggregation of GNP by sodium ions. Both aggregation and dispersion of GNP are shown

Developments in nanotechnological approaches have established a vision for the functionalization of nanomaterials for various purposes. In contrary to most nanomaterials, metal nanoparticles have garnered particular interest in scientific research due to their exceptional bio-recognition properties in sensing applications [1, 2]. Further exploration of metal nanoparticles has brought attention to the abilities of gold nanoparticles (GNP) due to their electrical and optical properties and due to their low cytotoxicity and inert nature [3]. GNP has been inculcated into various technologies encompassing photovoltaics, biomedicine, and chemical and biological catalysis. The fine tuneable nature of GNP also contributes to this as their chemical surface, size, and structure can be adjusted as intended. A copious range of functionalization can be implemented including peptide and amino acid conjugation, polymer attachment, and oligonucleotide coating [4–7]. Gold is charge neutral in its original state; however, for the ease of mobility and supply, GNP is contained within a capping agent that produces a weak binding effect on the surface of the GNP. Conventional capping agents utilized such as citrate and amino acid will respectively charge the surface of the GNP negatively and positively. In the controlled assembly and disassembly of GNP, balanced attractive and repulsive forces will determine aggregation and dispersion [8]. These changes can be easily analyzed by using a simple colorimetric assay system. In its original state, GNP is dispersed in orientation and exhibits a visually noticeable red color. The similar charges on the surface of the GNP will facilitate the repulsion between each particle. Hereby, GNP illustrates a wavelength of 520 nm under plasmon excitation. The ionic presence in the GNP colloidal solution will be charge neutralized when it reacts with a salt solution e.g., NaCl. The GNP will experience repulsion limitation whereby the loss of surface charge agglomerates the GNP to each other (Fig. 1). With microscopic analysis, the GNP is seen to aggregate towards each other under attractive forces. This causes a change from red color to purplish and a shift of wavelength to around 630 nm due to the induction of aggregation [6]. Due to the ease of visibility in the color changes of the colorimetric assay of GNP to demonstrate the variation in orientation, GNP has been proven to be advantageous. Attributing to this factor, the colorimetric assay analysis can be easily done via naked eye observation and without the requirement for costly high-end equipment. This analysis can also be conducted by personnel without highly skilled or technical background and sans prior training. The application of variations the assembly and disassembly of GNP is made evident by two concepts that have been studied in this research.

In this study, GNP was conjugated with poly(ethylene glycol) (PEG) to prevent the phenomenon of biofouling which is the assembly of undesired biological components on the sensing surface that leads to signaling sensitivity reduction. PEG functions for the inhibition of GNP aggregation due to non-specific reaction. The optimum conditions for conjugation such as type of GNP and type and concentration of PEG were studied. To show the variation in the binding of PEG with biomolecules, interaction between factor IX and factor IX-bp is shown, as factor IX-bp is a vital anticoagulant for excessive coagulation prevention. GNP assembly and disassembly concept was utilized for the study of molecular level interaction of these molecules. The optimum conditions that were investigated consisted of the size of GNP and concentrations of NaCl, factor IX, and factor IX-bp.

## Methods

### Materials

GNP and gold nanorods (GNR) in our study were purchased from NanoC Sdn. Bhd, Selangor, Malaysia. The analysis on PEG included polyoxyethylene (methoxy-PEG-(CH_2_)_2_-SH) with molecular weight 2000 and 5000, and poly(ethylene glycol)-*b*-poly(acrylic acid) with molecular weight of 2000. All three were supplied by Sunbright, CA, USA. Factor IX, a homologous protein containing N-terminal *γ*-carboxyglutamic acid was bought from American Diagnostica (Stamford, CT, USA), while factor IX-bp was prepared by the purification of snake venom of *Trimeresurus flavoviridis*. In addition, NaCl was bought from Avantor Performance Materials, Inc. in Pennsylvania, USA.

### Optimal Concentration of NaCl for the Aggregation of GNP

This experiment was done to identify the minimum concentration of NaCl for aggregation of GNP to occur. The red color of GNP would turn into blue or purple in the presence of NaCl. In this experiment, the different concentrations of NaCl which comprised of 0, 30, 60, 125, 250, and 500 mM were prepared by using serial dilution, and they were added to a constant volume of GNP. The appearances of color were recorded and compared.

### GNP Aggregation and Dispersion Analysis

A study was conducted on three different samples of gold namely, GNP of sizes 10, 15, and 30 nm. The readily dispersed state of the GNP samples was observed by Sigma 300 field emission scanning electron microscope (FESEM) analysis (Carl Zeiss Sdn. Bhd., Selangor, Malaysia). Ten microliters of each sample was dropped onto separate silicon wafers and left to dry in a fume hood for about 3 to 4 h until there was no liquid residue before FESEM analysis. The shapes of the particles were observed and analyzed. To observe the aggregation of GNP, a NaCl serial titration method was employed. Six Eppendorf tubes were filled with 9 μl of GNP of 10 nm. Tubes 1 to 5 were filled with 1 μl of H_2_O each, and the sixth tube was left as a control. Tubes 6 and 5 were filled with 1 μl of NaCl and shaken from a top to bottom motion to ensure thorough mixing. A micropipette was then used to extract 1 μl of the solution from tube 5 to be inserted into tube 4. This is followed by the top to bottom shaking. The serial dilution is continued right up to tube 2, and 1 μl of the solution from tube 2 was discarded for volume control. The color-turning point from red to purple was observed and recorded to identify optimum NaCl concentration for GNP aggregation. The aggregation and dispersion of GNP was monitored by UV-visible spectrophotometer by scanning from 400 to 800 nm.

### PEG Conjugation on Gold Particles

To compare the efficacy of spherical GNP versus GNR, the serial dilution method as in the aggregation and dispersion study was repeated with GNR. Six Eppendorf tubes were filled with 9 μl of GNR of 55 nm followed with the consecutive serial dilution steps as stated above. The optimum concentration for change of color was then observed between GNP and nanorods to identify which of the two required the lesser concentration of NaCl for aggregation.

### Type and Concentration of PEG for GNP Conjugation

Three types of PEG were studied which were polyoxyethylene (methoxy-PEG-(CH_2_)_2_-SH) with molecular weight 2000 and 5000 in addition to poly(ethylene glycol)-*b*-poly(acrylic acid) with molecular weight of 2000. Firstly, stock solutions were prepared for each of the PEG samples that were of concentration 1.6 mg/1000 μl of water whereby the weight of the PEG samples were measured with XA 100/2X weighing balance (RADWAG Wagi Elektroniczne, Radom, Poland). Then, six Eppendorf tubes were filled with 2.5 μl of distilled water except tube 6. Next, 7.5 μl of 10 nm GNP was inserted into each of the tubes with a micropipette. This step was followed by a serial dilution method. Prepared with polyoxyethylene (methoxy-PEG-(CH_2_)_2_-SH) with molecular weight 2000, 2.5 μl of the stock was inserted into tube 6 as a control. The same was done with tube 5. After thorough agitation, 2.5 μl of the solution from tube 5 was extracted and transferred into tube 4. This step was repeated until serial dilution is performed up to tube 2. A solution of 2.5 μl was extracted from tube 2 after the serial dilution to make the volumes constant. Finally, 0.5 μl of 5 M NaCl was dropped into each of the tubes and shaken again from top to bottom. The resulting colorimetric assay was observed, and the turning point of the color from purple to red state was observed and recorded to identify the optimum concentration of PEG. This experiment was then repeated with polyoxyethylene (methoxy-PEG-(CH_2_)_2_-SH) with molecular weight 5000 and poly(ethylene glycol)-*b*-poly(acrylic acid) with molecular weight of 2000.

### Optimization of Size of GNP in Nanometer

The effect of GNP sizes on the rate of appearance of purple color and the concentration of NaCl to be used were studied. The solutions of GNP with different sizes which were 10, 15, and 30 nm were prepared. These tubes were titrated with different concentrations of NaCl, and the turning point of color change was recorded.

### Effect of Concentration of Factor IX on the Aggregation of GNP

Firstly, different concentrations of factor IX which comprised of 0, 2320, 3480, 4640, and 5800 nM were prepared by using serial dilution. Then, the factor IX prepared was added to GNP solutions, respectively, followed by the addition of a constant concentration of NaCl. The color changes of GNP were recorded and compared.

### Interaction Between Factor IX and Different Concentrations of Factor IX-bp

Different concentrations of factor IX-bp which comprised of 0, 40, 60, 80, and 100 nM were prepared. First, factor IX was added to the GNP solutions, respectively. Subsequently, the factor IX-bp prepared was added to GNP solutions, followed by the addition of a constant concentration of NaCl. The color changes of GNP were recorded and compared.

### Interaction Between Thrombin and Different Concentrations of Factor IX-bp

The first step of this experiment was the preparation of two different concentrations of factor IX-bp, comprising of 0 and 100 nM. Then, thrombin (5.8 μM) was added to the respective GNP solutions. After that, the factor IX-bp prepared was added to the GNP solutions, followed by the addition of a constant concentration of NaCl. The intensity of color was recorded and compared.

## Results and Discussion

**Fig. 2:**
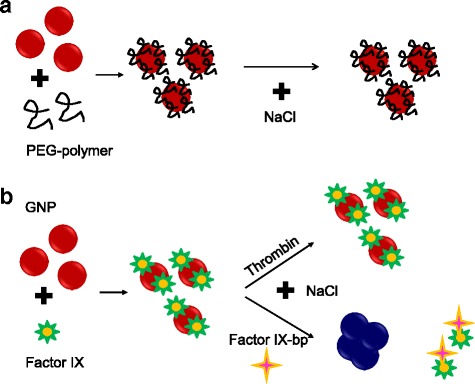
Proposed study to analyze the assembly and disassembly of molecules on GNP. **a** PEG-mediated GNP dispersion. PEG prevents the aggregation due to strong attachment on the GNP. **b** interaction of human clotting factor IX and factor IX-bp on the GNP. Interactions revealed by addition of NaCl is shown

GNP has the mechanism to spontaneously assemble and disassemble the molecules on it, due to electrostatic attraction, covalent binding, and hydrophobic absorption, which can be evaluated in the presence of NaCl (Fig. 2a) [9, 10]. To analyze and prove this mechanism, we considered two kinds of molecules were used to display the variations in the assembly processes namely, PEG-polymers which facilitates the non-fouling of GNP in sensing application. The second analysis involved the study of factor IX and factor IX-bp interaction. In the polymer case, due to strong interaction with GNP, an irreversible immobilization that can be applied in the prevention of biofouling was expected, whereas in the latter case, two important proteins playing a vital role in the blood clotting pathway were chosen to elucidate the different behaviors of proteins from the polymers. By optimizing these two different assembly processes, they can be implemented in the high-performance sensor developments.

PEG-polymers have been attested in sensing applications due to their capability to enhance sensitivity and specificity. Polymers also highly favor the proper aligning of biomolecules when co-immobilized [4, 7, 11, 12]. On the other hand, due to its ability to interact with other clotting factors, which are comprised of factors VIIIa, Va, XIa, and VIIa, the vital role of factor-IX cannot be ignored. Homologous protein factor-IX contains N-terminal Gla domain, which is vital for the anchorage of protein to the phospholipid membrane. Gla does not only aid the localization of protein to the injury site, but it also provides a proper position for the protein conduct interaction with factors [13, 14]. Lack of factor-IX in hemostatic system will lead to X-linked bleeding diathesis and hemophilia B. Factor IX-bp comprises of 129 and 123 amino acids for subunits A and B, respectively, which acts to impede the interaction between Gla domains, phospholipids, and the seat of the overall coagulation process. This ultimately leads to anticoagulation effect by inhibiting activation of prothrombin. GNP-based assay will be used as a system to study the interaction between factor IX and factor IX-bp and to compare the interaction between thrombin and factor IX-bp (Fig. 2b).

### Optimal Concentration of NaCl for the Aggregation of GNP

**Fig. 3:**
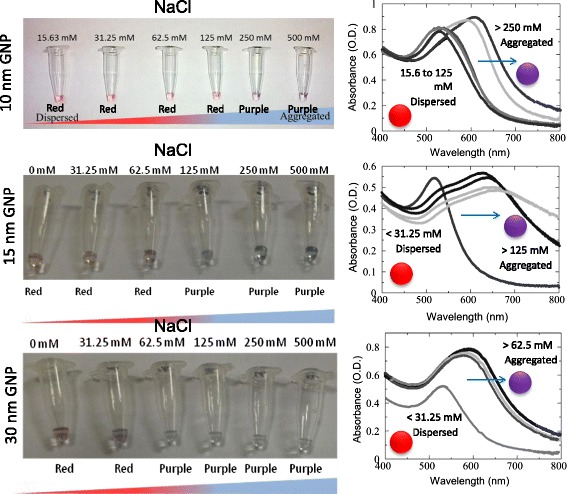
Dispersion and aggregation of GNP. Color Changes of GNP with different concentrations of NaCl. Both visible and UV-visible spectroscopic images are shown. Spectra are showing the changes from a *blue* to *red* shift. Shifts are indicated by *red* and *purple spheres*. An *arrow* indicates the direction of the shift. One optical density of GNP has been used

Normally, surface plasmon resonance (SPR) on the surface of GNP is localized and free electrons on the surface of GNP which had the same frequency with incident light resonate. SPR is the stage of excitation of surface plasmon (oscillating charge between two material’s interfaces) by light when there is an occurrence of total internal reflection. On the other view, the electrostatic repulsion between nanoparticles was induced by citrate ions on the GNP surface, stabilizing GNP against aggregation. Nevertheless, the frequency of oscillation of free electrons on the interface could be changed by adsorption of biomolecules. With the absence of NaCl, a red color solution appeared due to SPR adsorption peak at 520 nm. However, after adding of NaCl, a cross-linking between Na^+^ ion and ligands on GNP was formed. The electrostatic repulsion between GNP was disturbed by NaCl and positively charged sodium ion interacted with the negatively charged ions on the surface of GNP, giving a blue or purple color due to shifting of SPR adsorption peak to 630 nm [15]. Referring to Fig. 3 (left panel), when size 10 nm of GNP was used, the optimal concentration of NaCl (color-turning point) was 250 mM. When the concentration of NaCl below 250 mM was added, the color remained red as the aggregation of GNP was not completed. There were extra spaces available for Na^+^ to bind on the GNP surface and the citrate ions on the surface. When NaCl concentrations of 250 mM or above were added, the GNP aggregated completely. All citrate ions on the surface of GNP was bond by Na^+^ added and SPR absorption peak shifted to 630 from 520 nm, giving the purple color to the solution (Fig. 3; right panel).

### GNP Aggregation and Dispersion Analysis

**Fig. 4:**
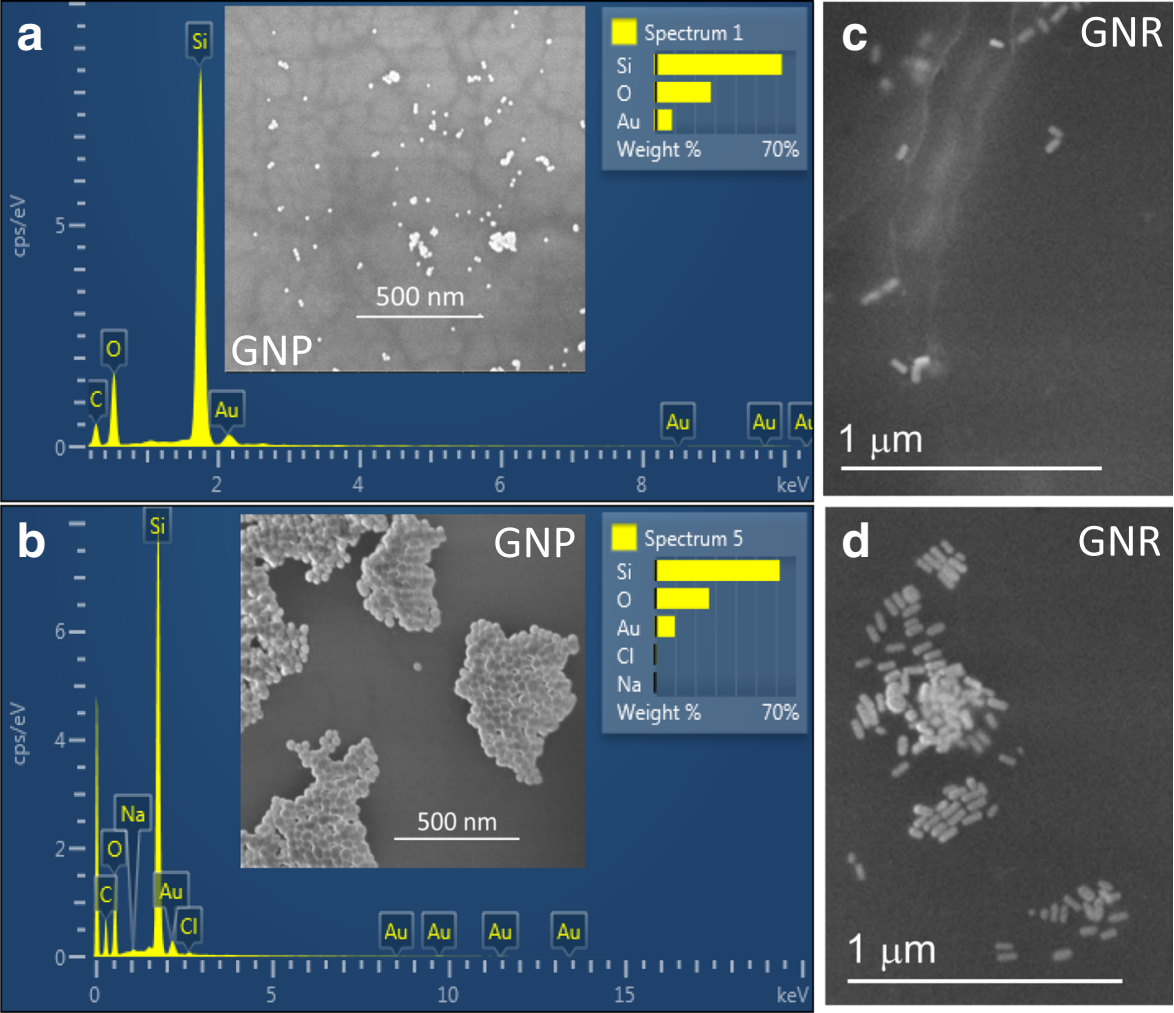
FESEM readily dispersed and aggregated states of GNP and GNR. **a** The scanned image showing the peaks of respective molecules in dispersion condition. Figure *inset* displays FESEM image. **b** The scanned image showing the peaks of respective molecules in aggregated condition. Na and Cl could be seen only with the aggregated state. Figure *inset* displays FESEM image. **c** Image showing GNR in dispersion. **d** Image showing GNR in aggregation. One optical density of GNP has been used

In this study, four samples of gold were used namely GNP of sizes 10, 15, and 30 nm. The remaining one was GNR of size 55 nm. From the FESEM analysis, it can be observed that the orientation of the GNP and GNR is aligned with theoretical information (Fig. 4a–d). When received from the supplier, both GNP and GNR were conventionally capped with capping agents [16]. Therefore, it is in a dispersed state as the similar charges between the particles tend to repel each other. When GNP is titrated with a salt such as NaCl, the GNP will be neutralized and its readily dispersed state changed into aggregated state because of the limitation in interparticle repulsion. According to the colorimetric assay observation as seen in Fig. 3, the color of the gold nanoparticles also exhibit a shift from red (dispersed state; blue shift) to purple (aggregated state; red shift). Figure 4a, b) shows the GNP under FESEM analysis, and these images clearly display the dispersion and aggregation of GNP. The peak profiles from SEM images also attest that sodium (Na) and chloride (Cl) peaks are available with aggregated state.

### PEG Conjugated Gold Particles

According to the results, the turning point of the GNP from red to purple is at the concentration of 250 nM (NaCl). The turning point of the GNR from red to purple could not be vividly observed (Fig. 4c, d). This might be attributed by the fact that GNR would require a higher concentration of NaCl to induce its aggregation. To facilitate efficient following reactions, the GNP is chosen over GNR due to a more visible reaction with lesser concentration of salt. Therefore, it also implies that in the following reactions, the observation of the effects of PEG conjugation may be done in the minimum presence of salt with GNP compared to GNR. The variation in the surface properties and geometry between the GNP and GNR is due to functionalized or reacted by different methods, changes in structure, thermodynamic properties, and activity. GNP is also favored in the following experiments due to a number of reasons. Proteins that have been reacted with GNP have shown evidence of retaining their native properties and structure when it is functionalized with GNP. Contrastingly, the GNR functionalization with protein which is entropically favored may cause a loss of secondary and tertiary structures in protein if the water bound within is released at a large amount.

#### Type and Concentrations of PEG for GNP Conjugation

**Fig. 5:**
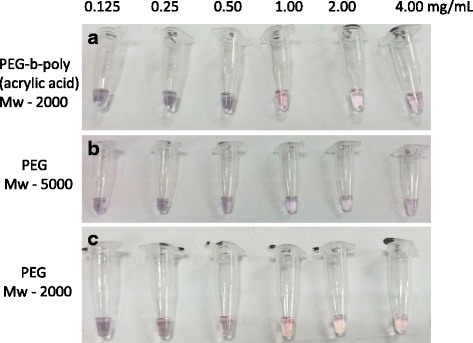
Dispersion and aggregation of GNP in the presence PEG-polymers. **a** Poly(ethylene glycol)-*b*-poly(acrylic acid) with Mw 2000, **b** polyoxyethylene with Mw 2000, and **c** polyoxyethylene with Mw 5000. Color Changes of GNP with the addition of NaCl. One optical density of GNP has been used

From the colorimetric assay of three types of PEGs, it can be noted that poly(ethylene glycol)-*b*-poly(acrylic acid) with Mw 2000 portrayed the most vivid change in color from red to dark purple at the concentration of 1 mg/ml (Fig. 5a). This is followed by polyoxyethylene with Mw 2000 which showed only a gradual change in color from the concentration of 1 mg/ml and lower (Fig. 5b). This suggests that concentrations of 1 mg/ml of both of these PEGs are required to inhibit the aggregation of GNP and maintaining the red dispersed state. However, polyoxyethylene with Mw 5000 immediately showed a change to the color purple from its concentration of 4 mg/ml (Fig. 5c). This means that a higher concentration of this PEG was required for conjugation with PEG to withstand the change from dispersed red state to aggregated purple state. Thus, this is uneconomical and inefficient for the experiment. Poly(ethylene glycol)-b-poly(acrylic acid) can be chosen due to its stability and more branched structure that provides better coverage.

#### Optimization of Size of GNP in Nanometer

By increasing the size of GNP, the interparticle distance is expected to be decreased. Consequently, the peak of optical absorbance for GNP shifted to a large wavelength and a visible color change of the GNP solution from red to purple [17]. When a larger size of GNP is used, the interparticle distance became smaller and the frequency of oscillation of free electrons on the interface could be changed easily by adsorption of Na^+^. Whereas, when a smaller size of GNP is used, the interparticle distance between nanoparticles was large and more Na^+^ from NaCl was needed to aggregate the GNP. Three different experiments were done by using different sizes of GNP which were 10, 15, and 30 nm. By referring to Fig. 3, when size 10 nm of GNP was used, the optimal concentration of NaCl (color-turning point) was 250 mM. When NaCl concentrations below 250 mM were added, the color remained red as the aggregation of GNP was not completed. There were extra spaces available for Na^+^ to bind on the GNP surface and the citrate ions on the surface. When NaCl concentrations of 250 mM or above were added, the GNP aggregated completely. All citrate ions on the surface of GNP was bond by Na^+^ added and SPR adsorption peak was shifted to 630 from 520 nm, giving the purple color to the solution.

However, from Fig. 3, when size 15 nm of GNP was used, the optimal concentration of NaCl (color-turning point) was 125 mM. When NaCl concentrations below 125 mM were added, the color remained red as the aggregation of GNP was not completed. There were extra spaces available for Na^+^ to bind on the GNP surface and the citrate ions on the surface. When NaCl concentrations of 250 mM or above were added, the GNP aggregated completely. According to Fig. 3, the optimal concentration of NaCl (color-turning point) was 62.5 mM when size 30 nm of GNP was used. The color remained red when NaCl concentrations below 62.5 mM were used. This was because the aggregation of GNP was not completed. When NaCl concentrations of 250 mM or above were added, the GNP aggregated completely. By comparing the three experiments, the results showed that the lowest concentration of 62.5 mM NaCl was needed to crosslink the GNP particles and form aggregation of GNP when 30 nm of GNP used. Only 62.5 mM of NaCl was required to induce aggregation by the complete binding of Na^+^ and citrate ions on the GNP surface when 30 nm of GNP size was used. The lower the concentration of NaCl needed, the better the size of GNP used. In conclusion, the 30 nm of GNP size was chose as the optimal size for the next experiments.

### Study on the Interaction Between Factor IX and Factor IX-bp

#### Effect of Concentrations of Factor IX on the Aggregation of GNP

**Fig. 6:**
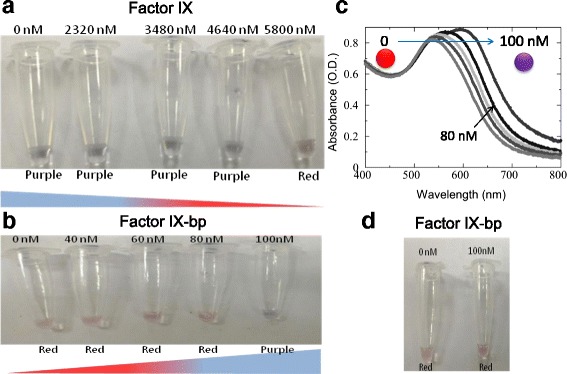
Dispersion and aggregation of GNP in the presence biomolecule. **a** In the presence of different factor IX concentration. **b** Interaction of factor IX-bp on factor IX immobilized GNP. **c** UV-visible spectroscopic analysis showing the changes from a *blue* to *red* shift. Shifts are indicated by *red* and *purple spheres*. An *arrow* indicates the direction of the shift. Sensitivity was ~80 nM as indicated. **d** Interaction of factor IX-bp on thrombin immobilized GNP; 5.8 μM of thrombin was immobilized on the GNP. Color changes of GNP was tested with the addition of NaCl. One optical density of GNP has been used

Figure 6a showed that in the presence of factor IX, a binding between factor IX and GNP was formed. As a result, there was no extra space at ligands on GNP and Na^+^ was unable to bind to the GNP. Consequently, cross-linking between Na^+^ and GNP could not be formed and the aggregation was inhibited as Na^+^ could not bind to the citrate ions on the surface of GNP. The electrostatic repulsion between nanoparticles was induced by citrate ions on the GNP surface, stabilizing GNP against the aggregation, giving the red color to the solution [8]. In this experiment, the purple color appeared in the first four concentrations (until 4640 nM) indicating that there was aggregation of GNP. These results may be due to incomplete binding between ligands on the GNP and factor IX. From Fig. 6a, the minimum concentration of factor IX needed to prevent aggregation of GNP was 5800 nM as red color appeared. Initially, the GNP turned purple as all the surface of the GNP was bonded by Na^+^ and the electrostatic repulsion between GNP was disturbed by NaCl, giving blue or purple color due to shifting of SPR adsorption peak to 630 from 520 nm.

#### Interaction Between Factor IX and Different Concentrations of Factor IX-bp

Two sets of experiments were done to find out the minimum factor IX-bp concentration needed to recover the original state of ligand on GNP to be occupied by NaCl. Besides that, this experiment was done to investigate the minimum concentration of factor IX-bp needed to prevent coagulation in blood when there was 5800 nM of factor IX. Figure 2 showed the mechanism of the interaction between factor IX and factor IX-bp on GNP. Initially, the red color of GNP remained as the dispersion was inhibited by 5800 nM of factor IX. After factor IX-bp was added, factor IX-bp would bind to factor IX, and thus, there were spaces available for Na^+^ ions binding. When there was complete removal of factor IX by factor IX-bp, frequency of oscillation of free electron on the interface of GNP was changed by adsorption of Na^+^. Consequently, positive charged sodium ion interacted with the negative charged ions on the surface of GNP, giving blue or purple color due to the shifting of SPR adsorption peak at 630 nm.

The first set of experiment was done by using 0, 4, 6, 8, and10 nM factor IX-bp, which indicated that the concentration below 10 nM was insufficient to prevent coagulation when there was 5800 nM of factor IX in blood. This was because there was no complete removal of factor IX by factor IX-bp, and thus, sodium ion could not bind to citrate ion on the surface of GNP. Consequently, the SPR adsorption peak remained at 520 nm, giving red color to GNP. The latter set of experiment was done by using 0, 40, 60, 80, and 100 nM of factor IX-bp. The result in Fig. 6b showed 100 nM of factor IX-bp was needed to prevent coagulation with 5800 nM of factor IX. When the concentration below 100 nM was used, the removal of factor IX was incomplete. However, with the UV-visible spectroscopic analysis, we can monitor a clear peak shift at ~80 nM, indicates the sensitivity range of factor IX to interact with factor IX-bp (Fig. 6c). The purple color formed at the last GNP solution indicated that all factor IX in the GNP interacted with factor IX-bp, so that there were spaces at ligand on GNP for Na^+^ causing a shift of the SPR adsorption peak from 520 to 630 nm.

#### Interaction Between Thrombin and Different Concentrations of Factor IX-bp

Thrombin is an enzyme (protein) that plays an important role in blood coagulation system, which would convert fibrinogen to fibrin [18]. Normally, thrombin would bind to GNP, and thus, the surface of GNP would be occupied, leading to less or no space available for the binding of Na^+^ as shown in Fig. 6d. This experiment was done to indicate there was no interaction between thrombin and factor IX-bp. When there was interaction between thrombin and factor IX-bp, the thrombin attached on the surface would be removed by factor IX-bp and Na^+^ could bind to the surface and the SPR adsorption peak was shifted from 520 to 630 nm. When there was no interaction between thrombin and factor IX-bp, the red color would remain. The results showed that the red color remained constant either with or without the presence of factor IX-bp. The red color indicated there was interaction between thrombin and GNP and this would cause the space of ligand on GNP to be occupied. After factor IX-bp was added, the result remained same, as factor IX-bp was unable to remove thrombin from GNP. This result proved that factor IX-bp would only bind to factor IX specifically but not with thrombin.

## Conclusions

The study of variations in spontaneous and controlled assembly and disassembly of GNP has led to several outcomes. It has been made evident that in its original state, GNP retains a dispersed state and the color is red. However, once reacted with NaCl, it changes into an aggregated orientation of the color purple. Firstly, in the study of PEG conjugated GNP, it was found that PEG minimized non-specific reactions as GNP remains in dispersed state. The GNP of spherical shape was found to be more effective than GNR due to minimum concentration of NaCl required for reaction. Besides, the best suited PEG was known to be poly(ethylene glycol)-b-poly(acrylic acid) with Mw 2000 of concentration 1 mg/ml. In addition, in the analysis of interaction between factor IX and factor IX-bp by GNP-based assay, it was proved that factor IX would be desorbed from GNP by factor IX-bp, which then provided space for Na^+^ ion binding on the GNP surface and purple color was formed. However, there were some variables that might influence the color changes of GNP solution, which including the concentration of NaCl, factor IX and factor IX-bp used, and the size of GNP. The experiment proved that the optimal concentration of NaCl for aggregation was at least 250 mM for all sizes of GNP. Besides, the optimal size of GNP used was 30 nm. The concentration of 5800 nM of factor IX was proved to be optimum for aggregation of GNP. Furthermore, ~80 nM of factor IX-bp was needed to remove factor IX from the surface of GNP. Besides, it was proved that thrombin could not interact with factor IX-bp and it could not be used as a substitute. The study shown here carries the novel method using unmodified GNP to analyze the spontaneous assembly and disassembly of molecules as a sensor and biofouling can be prevented by PEG-polymers.
